# Liver proteomics identifies a disconnect between proteins associated with de novo lipogenesis and triglyceride storage

**DOI:** 10.1016/j.jlr.2024.100687

**Published:** 2024-10-25

**Authors:** Lewin Small, Tuong-Vi Nguyen, Mark Larance, Darren N. Saunders, Andrew J. Hoy, Carsten Schmitz-Peiffer, Gregory J. Cooney, Amanda E. Brandon

**Affiliations:** 1 School of Life and Environmental Sciences, Charles Perkins Centre, Faculty of Science, The University of Sydney, Sydney, NSW, Australia; 2 Garvan Institute, Sydney, NSW, Australia; 3 School of Medical Sciences, Charles Perkins Centre, Faculty of Medicine and Health, University of Sydney, Sydney, NSW, Australia

**Keywords:** liver, de novo lipogenesis, steatosis, proteomics, triglycerides, semipurified diet

## Abstract

De novo lipogenesis (DNL) has been implicated in the development and progression of liver steatosis. Hepatic DNL is strongly influenced by dietary macronutrient composition with diets high in carbohydrate increasing DNL while diets high in fat decrease DNL. The enzymes in the core DNL pathway have been well characterized; however, less is known about other liver proteins that play accessory or regulatory roles. In the current study, we associate measured rates of hepatic DNL and fat content with liver proteomic analysis in mice to identify known and unknown proteins that may have a role in DNL. Male mice were fed either a standard chow diet, a semipurified high starch or high-fat diet. Both semipurified diets resulted in increased body weight, fat mass, and liver triglyceride content compared to chow controls, and hepatic DNL was increased in the high starch and decreased in high fat-fed mice. Proteomic analysis identified novel proteins associated with DNL that are involved in taurine metabolism, suggesting a link between these pathways. There was no relationship between proteins that associated with DNL and those associated with liver triglyceride content. Further analysis identified proteins that are differentially regulated when comparing a nonpurified chow diet to either of the semipurified diets, which provide a set of proteins that are influenced by dietary complexity. Finally, we compared the liver proteome between 4- and 30-week diet-fed mice and found remarkable similarity suggesting that metabolic remodeling of the liver occurs rapidly in response to differing dietary components. Together, these findings highlight novel proteins associated with hepatic DNL independently of liver fat content and suggest rapid liver metabolic remodeling in response to dietary composition changes.

De novo lipogenesis (DNL), the synthesis of fatty acids from non-lipid sources (largely carbohydrates, acetate and amino acids) has been implicated in the development and progression of liver steatosis and associated diseases (nonalcoholic fatty liver disease [NAFLD]) (1, 2, 3). Although DNL makes a relatively small contribution to circulating triglycerides (TGs) in individuals with low liver fat (∼3–10%) (2, 4, 5), this can increase substantially in individuals with liver steatosis (15–26%) (2, 5, 6), without increases in the contribution of other sources of TG (fatty acids from adipose tissue lipolysis or digestion). Additionally, rates of DNL are substantially increased in humans who consume carbohydrate-rich diets (7, 8) or an unbalanced diet elevated in simple sugars (9). DNL primarily occurs in the liver and adipose tissue and in humans (4) and mice (10) hepatic lipogenesis is more responsive to increases in dietary carbohydrate and occurs at higher rates compared to that in adipose tissue.

The enzymes in the core DNL pathway, as well as the transcription factors that regulate their transcription, have been well characterized. Cytosolic acetyl-CoA, derived from citrate generated in the mitochondrial tricarboxylic acid cycle, or acetate, is used as a substrate by the enzyme acetyl-CoA carboxylase to generate malonyl-CoA, in an ATP-dependent reaction. The FAS enzyme complex initiates fatty acid synthesis by the condensation of acetyl-CoA and malonyl-CoA. The fatty acid is then extended by the sequential addition of acetyl groups from malonyl-CoA, in a NADPH-dependent reaction, resulting in the 16-carbon, saturated fatty-acid palmitoyl-CoA (11, 12). DNL enzymes are transcriptionally regulated by the transcription factors SREBP and ChREBP, which in response to insulin (SREBP) or levels of glycolytic intermediates (ChREBP), translocate to the nucleus and bind to SRE (SREBP) or ChORE (ChREBP) motifs on DNA, driving transcription (13). Less is known about other proteins that play accessory roles to DNL or are involved in posttranscriptional regulation. For example, the nuclear protein, thyroid hormone-responsive SPOT 14 homolog (Thrsp) is thought to regulate lipogenesis, and overexpression increases the expression of core DNL proteins and hepatic TG (14); however, how it exerts these effects is largely unknown.

Mouse models have been pivotal to the understanding of the enzymes involved in the DNL pathway and its regulation. The two main types of mouse models used to investigate DNL are either transgenic (e.g., knockdown or overexpression of transgenic SREBP (15) or ChREBP (16)) or dietary (17, 18). Dietary models provide a more physiological view of how DNL is regulated by different macronutrients. Transcriptomic or proteomic studies of NAFLD often compare mice that differ in both liver fat content as well as rate of lipogenesis (19, 20, 21); therefore, it is hard to parse the contribution of one compared to the other. Additionally, preclinical studies often compare an obesogenic semipurified diet with a nonpurified chow control diet (22, 23, 24, 25, 26, 27); therefore, it is not clear if protein abundance differences are due to differences in liver fat, lipogenesis, or the metabolism of other dietary components.

In a recent study, our group published a dietary model in which we compare mice fed a standard laboratory chow to those fed a palatable semipurified high-starch diet or high-fat diet (10). High-starch mice displayed an equivalent increase in body weight, fat mass, and liver fat content as high-fat mice compared to chow mice; however, rates of hepatic DNL were elevated in high-starch mice, intermediate in chow mice, and low in high-fat mice, whereas adipose tissue DNL was not affected by diet. We thought that this would be an ideal model to investigate proteins in the DNL pathway and how they react to alterations in diet in a nonbiased way; as in this model, liver TG content and DNL are dissociated.

In the current study, rates of DNL, TG levels, and liver protein abundance were assessed in livers from mice fed the above diets for a period of 4 weeks. We then identified proteins that associated with either the rate of DNL or TG content in the liver. Further analysis identified proteins that were differentially regulated by comparing a nonpurified chow diet to either of the semipurified diets and comparing the effect of short- and long-term diet feeding on the liver proteome. To the best of our knowledge, this is the first proteomic investigation of proteins that associate with liver lipogenesis using an actual measure of the rate of hepatic lipogenesis and complements existing studies that utilize genetic models of DNL regulation (e.g., SREBP knockout and transgenic mice (15)).

## Materials and Methods

### Animal husbandry and procedures

#### Ethics

All experimental procedures performed were approved by the Garvan Institute/St Vincent’s Hospital Animal Ethics Committee and were in accordance with the National Health and Medical Research Council of Australia’s guidelines on animal experimentation (protocol number: 14_07).

#### Dietary intervention

Male C57BL/6J_Arc_ mice (8 weeks of age) were sourced from Animal Resource Centre (Perth, Australia), and after a 1 week acclimatization period, mice were randomly assigned to receive either a standard chow diet (6% fat, 23% protein, and 71% carbohydrate, by calories; Gordon Specialty feeds, Sydney Australia; energy density 13.0 kJ/g), a high-starch diet (Hi-ST; 22% protein, 57% carbohydrate, and 21% fat, by calories) or a lard-based, high-fat diet (Hi-F; 22% protein, 21% carbohydrate, and 57% fat, by calories), made in house and were available ad libitum. The exact composition of the semipurified test diets has been previously described (10). Mice were communally housed (4–5 per cage) in a temperature-controlled (29°C ± 0.5°C) and light-controlled (12 h light:12 h darkness cycle, 07:00–19:00 light) room with corn cob bedding.

Mice were fed for either 4 (n = 15 per diet) or 30 weeks (n = 10 per diet) prior to being euthanized. Body weights were measured weekly. In the 4-week cohort, fat mass was measured at 3 weeks of age using the EchoMRI-500 (EchoMRI LLC, Houston, TX) according to the manufacturers’ instructions, excluding body water. In the 30-week cohort, fat mass was measured at 16 weeks of age by dual X-ray absorptiometry using a PIXImus small-animal densitometer and associated software (PIXImus II; GE Medical Systems, Madison, WI).

Body weight, body composition, rates of DNL and liver TG content data from a subset of the 4-week cohort have been previously published (10). Data from the 30-week diet-fed cohort and all proteomic analyses have not been published elsewhere.

#### Assessment of DNL and TG level in liver

After 4 weeks of diet, the rate of DNL was measured. Briefly, at 8:00 am in the postprandial state, mice were given an intraperitoneal injection of 0.5 mCi of ^3^H_2_O in 200 μl of sterile saline. One hour later, animals were killed by cervical dislocation, and plasma and liver were collected. Tissue saponification and determination of ^3^H incorporation into fatty acids were conducted as previously described (28). Rates of liver DNL were determined by dividing tissue lipid ^3^H counts by the specific activity of ^3^H in plasma and correcting for time and tissue weight.

### Biochemical techniques

Liver TGs were extracted using the Folch method (29) and quantified as glycerol equivalents using an enzymatic colorimetric method (GPO-PAP reagent; Roche Diagnostics). Enzymatic activity assays and immunoblotting for validation of mass spectrometry-derived relative protein abundance were performed as previously described (10).

### Sample preparation for mass spectrometry-based proteomics of liver tissue

About 50 mg of frozen liver tissue was weighed out and lysed in 1 ml of 4% SDS, 100 mM NaCl, 20 mM sodium phosphate (pH 6.0), 10 mM NaF, 10 mM NEM, 10 mM sodium pyrophosphate, 2 mM sodium orthovanadate, and 60 mM sodium β-glycerophosphate containing cOmplete protease inhibitor EDTA-free (Roche), using an Ultra-Turrax T8 stick homogeniser (IKA®; Werke) to blend tissue before heating lysates to 65°C for 10 min (30). Lysates were then sonicated at 80% amplitude for 15:15 s pulses with 10 min total on-time at 20°C (QSONICA) before centrifugation at 18,000 *g* for 10 min at room temperature. The lipid layer was aspirated from the clarified lysate, and protein concentration was determined by BCA Total protein assay (Pierce). About 100 μg of protein was subjected to reduction with Tris(2-carboxyethyl)phosphine for 10 min at 65°C and alkylated with NEM for 30 min at room temperature. Samples were then processed for trypsin digestion and cleanup using the SP3 method (31). SP3 peptide eluates were then cleaned using SDB-RPS StageTips as described previously (32). Peptides were resuspended in 5% formic acid and stored at 4°C until acquired by LC-MS.

### LC-MS/MS and analysis of spectra

Using a Thermo Fisher Scientific U3000 nano-UHPLC, peptides in 5% (v/v) formic acid (injection volume 3 μl) were directly injected onto a 45 cm × 75 μm C18 (Dr Maisch, Ammerbuch, Germany; 1.9 μm) fused silica analytical column with a ∼10 μm pulled tip, coupled online to a nanospray ESI source. Peptides were resolved over a gradient from 5% acetonitrile to 40% acetonitrile over 120 min with a flow rate of 300 nl min^−1^. Peptides were ionized by electrospray ionization at 2.3 kV. Tandem mass spectrometry analysis was carried out on a Q-Exactive Plus mass spectrometer (ThermoFisher) using high-energy collisional dissociation fragmentation. The data-dependent acquisition method used acquired MS/MS spectra of the top 20 most abundant ions at any one point during the gradient. RAW data were analyzed using the quantitative proteomics software MaxQuant (version 1.5.7.0). This version of MaxQuant includes an integrated search engine, Andromeda. Peptide and protein level identification were both set to a false discovery rate of 1% using a target-decoy-based strategy. The database supplied to the search engine for peptide identifications contained both the mouse UniProt database downloaded on June 14, 2017, containing 59,594 protein sequence entries and the MaxQuant contaminants database. Mass tolerance was set to 4.5 ppm for precursor ions, and MS/MS mass tolerance was 20 ppm. Enzyme specificity was set to trypsin (cleavage C-terminal to Lys and Arg) with a maximum of two missed cleavages permitted. Deamidation of Asn and Gln, oxidation of Met, pyro-Glu (with peptide N-term Gln), and protein N-terminal acetylation were set as variable modifications. N-ethylmaleimide on Cys was searched as a fixed modification. We used the MaxLFQ algorithm for label-free quantitation, integrated into the MaxQuant environment.

### Proteomic data analysis

Proteomic data were filtered for contaminants, reversed matches, precursors identified only by site and proteins identified by less than two unique peptides. Label-free quantitation data were log transformed and median normalized by sample. Sample to sample correlation was performed on the intensity matrix to isolate samples with possible technical issues from mass spectrometry, six samples were excluded based on a mean correlation coefficient of under 0.9. Proteins were filtered out if they were quantified in less than eight mice in each of the three diet groups in the 4-week-fed cohort or six mice in each of the three diet groups in the 30-week-fed cohort unless they were determined to be missing not at random, which was determined by the percentage difference in missing values of greater than 60% between any two groups. Imputation of proteins missing not at random (seven proteins) was performed using a left censored method (QRILC) in the qFeatures package (https://github.com/RforMassSpectrometry/QFeatures) Multidimensional scaling (MDS) analysis was performed using limma (33), and another five samples were identified and excluded that were clear outliers by MDS analysis and were noted down to have muscle contamination during mouse dissection (supplemental Fig. S1A). Three mice did not have a value for lipogenesis due to technical issues. Limma (33) was used to determine differentially abundant proteins that correlated with a continuous phenotype (rate of DNL, TG content, and log2 transformed) or to compare between diets using the eBayes setting with trend = T, robust = T. *P* values were corrected for multiple hypothesis testing using the standard Benjamini-Hochberg correction in limma (false discovery rate [FDR]). When adjusting for TG content, TG content as a continuous variable was included in the linear model. An adjusted *P* value of < 0.05 was considered statistically significant. Gene overrepresentation analysis was performed using the clusterProfiler package (34) separately on upregulated and downregulated proteins compared to a background of all proteins quantified after filtering, looking at GO-BP (Gene Ontology-biological process) terms with default settings. Gene set enrichment analysis was performed using the clusterProfiler package, sorting protein lists with a ranking metric of -log10 pvalue ∗ sign(logFC), looking at GO-BP terms. An excel spreadsheet containing the statistics for all the proteomic comparisons is included in the supplemental data.

### Statistical analysis

Data are expressed as means ± SEM or individual points. Apart from proteomic data analyzed as described above, results were analyzed by a one-way ANOVA, two-way repeated measures ANOVA, or simple linear regression, as specified in the figure legends. If the ANOVA reached statistical significance, a Tukey post hoc test was used to test between diets. Statistical analysis was performed in GraphPad Prism software (Prism 10; La Jolla). Statistical significance was set at *P* < 0.05.

## Results

### Mice fed a semipurified high-starch diet have similar adiposity and liver fat content to mice fed a high-fat diet but substantially increased hepatic DNL

**Fig. 1:**
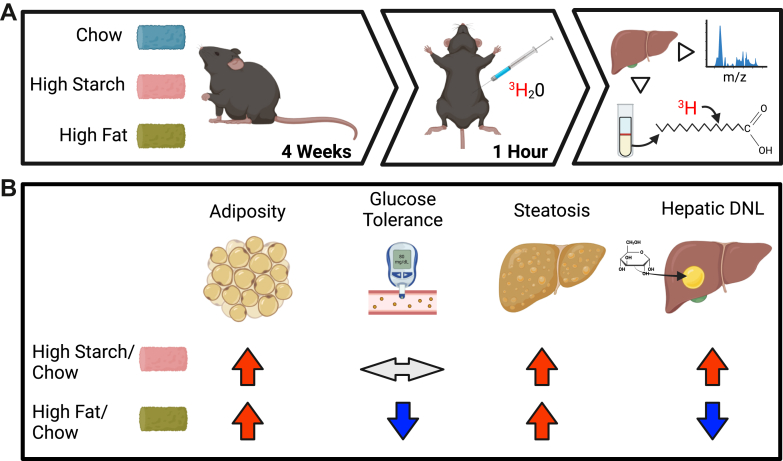
Physiological data from mice fed chow, a high-starch, or a high-fat diet. A: Schematic of experimental design where male C57BL/6J mice were fed one of three diets, standard laboratory chow or a semipurified high-starch or high-fat diet for a period of 4 weeks, and then, DNL was measured by injecting mice with ^3^H_2_O, sacrificing them 1 h later and then measuring ^3^H content in the liver fatty acid pool as well as measuring the liver proteome. B: A summary of the previously published metabolic parameters of high-fat and high-starch-fed mice compared to chow-fed mice (10).

Male C57BL/6J mice were fed either standard laboratory chow, a semipurified high-starch diet, or a semipurified high-fat diet from 8 weeks of age for a period of 4 weeks (Fig. 1A), during which time they were housed at thermoneutrality (29°C). As we previously reported (10), mice fed a high-starch diet had similar body weight, fat mass, and liver TG content to high-fat diet-fed mice, which were all significantly elevated above chow-fed mice (summarized in Fig. 1B). The rate of hepatic DNL was estimated by the incorporation of tritium into newly synthesized fatty acids in the liver (Fig. 1A). High-starch mice had greater rates of hepatic DNL, whereas high fat-fed mice had slower rates compared to chow-fed mice.

### Correlating protein abundance with lipogenesis identifies proteins with known and uncharacterized functions in the hepatic de novo lipogenic pathway

**Fig. 2:**
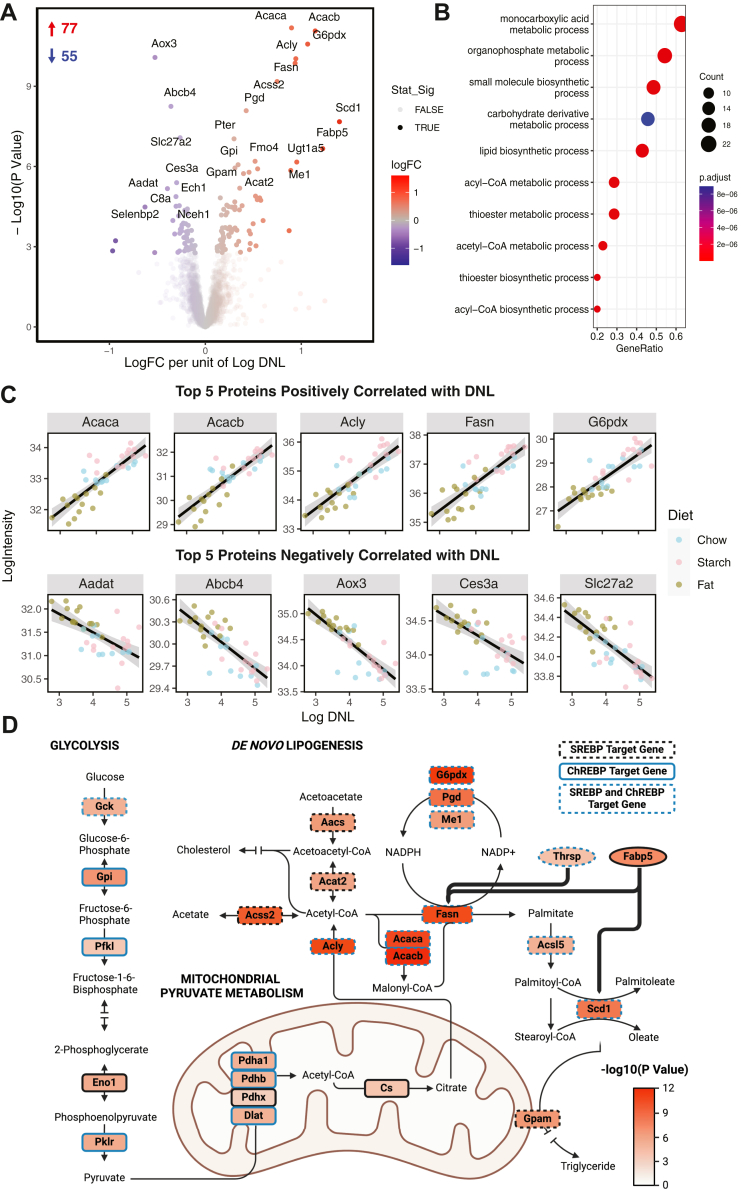
Proteins that associate with the rate of hepatic DNL. A: Volcano plot of liver proteins that positively (red) or negatively (blue) associate with the rate of hepatic DNL in mice. Transparent dots are proteins that did not reach the statistical significance threshold (FDR <0.05). B: Gene overrepresentation analysis (Gene Ontology-biological process [GO-BP]) of proteins that were significantly positively associated (FDR <0.01) with the rate of DNL over a background of all proteins quantified after filtering, color represents the adjusted *P* value of the term, dot size represents the counts within that term. C: The top five proteins that were either significantly positively or negatively associated with hepatic DNL plotting log2 intensity of the protein against log2 hepatic DNL (μmol ^3^H_2_O/h/g). D: Proteins that were significantly positively associated with rate of hepatic DNL plotted onto the DNL pathway and associated pathways, a blue border represents a known ChREBP target, and a dashed border represents a known SREBP target. Known SREBP target genes were included from supplemental Table S3 of the following article (54). Known ChREBP target genes were included from a combined list from Table 2 (55), Table 1 (56), and Table 2 (57) of their respective articles.

As high-starch and high-fat diet-fed mice had similar liver fat content, but substantially different rates of lipogenesis, this could be used to discriminate between proteins associated with liver fat from those associated with DNL. Therefore, we next correlated protein abundance with the rate of liver lipogenesis in each mouse, regardless of diet, due to substantial within-group variation for this measurement. Variation between samples and dietary groups in the proteomics data can be visualized in supplemental Fig. S1B, where mice from dietary groups clustered together; however, these clusters displayed substantial overlaps. We included proteins in our dataset if it was detected in at least eight mice per diet (unless determined missing not at random, see Materials and Methods section). From this, 3,751 unique proteins were identified, and their abundance was correlated to rate of hepatic DNL. As a result, 77 proteins had significant positive correlation with the rate of liver lipogenesis and 55 proteins had significant negative correlation (FDR <0.05, Fig. 2A). Gene overrepresentation analysis of proteins that positively associated with lipogenesis were enrichment of components of monocarboxylic acid metabolism, lipid biosynthesis, and acetyl-CoA metabolism pathways, as expected (Fig. 2B). Interestingly, there were no pathways enriched for proteins negatively associated with lipogenesis; however, the most significant negatively correlated proteins were involved in the metabolism of aldehydes (Aox3), carboxyl-esters (Ces3a), or are lipid transporters (Abcb4, Slc27a2). The top five positively and negatively correlated proteins with lipogenesis are depicted in Figure 2C, showing a tight correlation between protein abundance and lipogenesis. Changes in Acc, Fasn, Acly, Scd1, and G6pdx were further validated by immunoblotting (Acc, Fasn, and Scd1) or enzymatic activity assay (Acly and G6pdx) showing a good accordance between MS-derived abundance and immunoblotting/activity quantitation (supplemental Fig. S2). We next placed proteins that were significantly positively correlated with lipogenesis into the known DNL pathway (Fig. 2D). Confirming the effectiveness of this approach, every enzyme in the DNL pathway significantly positively correlated with the rate of hepatic lipogenesis. Central DNL enzymes (Acly, Acaca, Acacab, Fasn, and G6pdx) displayed the highest correlation between protein abundance and the rate of hepatic DNL. As well as core DNL enzymes, putative regulators of lipogenesis (Thrsp and Fabp), and multiple enzymes in glycolysis and mitochondrial pyruvate metabolism, responsible for converting glucose into cytosolic-acetyl-CoA to be used as a substrate in DNL were positively correlated with DNL. Figure 2D also highlights proteins that are known as SREBP and/or ChREBP target genes. Additionally, there were many proteins identified using this analysis that have no known role in DNL (Table 1), although some are SREBP target proteins, and many have roles in other lipid metabolic pathways.

**Table 1:** List of proteins that positively correlate with rate of hepatic DNL with no characterized role in the DNL pathway

Protein name	Gene symbol	Adjusted *P* value	Known TF
Phosphotriesterase related protein	Pter	2.8898E-05	
Flavin containing dimethylaniline monoxygenase 4	Fmo4	0.0001699	
UDP glucuronosyltransferase family 1 member A5	Ugt1a5	0.0001699	
Retinol dehydrogenase 11	Rdh11	0.00025579	SREBP
Glutamyl aminopeptidase	Enpep	0.0003299	
Glutathione-*S*-transferase Mu 2	Gstm2	0.00193334	
Lanosterol synthase	Lss	0.00225656	SREBP
Farnesyl diphosphate synthase	Fdps	0.0022977	SREBP
Phospholysine phosphohistidine inorganic pyrophosphate phosphatase	Lhpp	0.00255267	
Arsenite methyltransferase	As3mt	0.00318134	
Cytochrome B5 reductase 3	Cyb5r3	0.00318134	
Quinoid dihydropteridine reductase	Qdpr	0.00364807	
NHS like 3 (Kiaa1522)	Nhsl3	0.00367487	
Phosphate cytidylyltransferase 2, ethanolamine	Pcyt2	0.00509053	SREBP
Annexin A6	Anxa6	0.00509053	
Glutathione-*S*-transferase Pi 1 (BC021614)	Gstp3	0.0077855	
Ganglioside GM2 activator	Gm2a	0.00963129	
Tubulin folding cofactor E like	Tbcel	0.01224658	
Caspase 3	Casp3	0.01227007	
Sigma non-opioid intracellular receptor 1	Sigmar1	0.01227007	

### No overlap between proteins that correlate with DNL and those that correlate with TG content in the liver

**Fig. 3:**
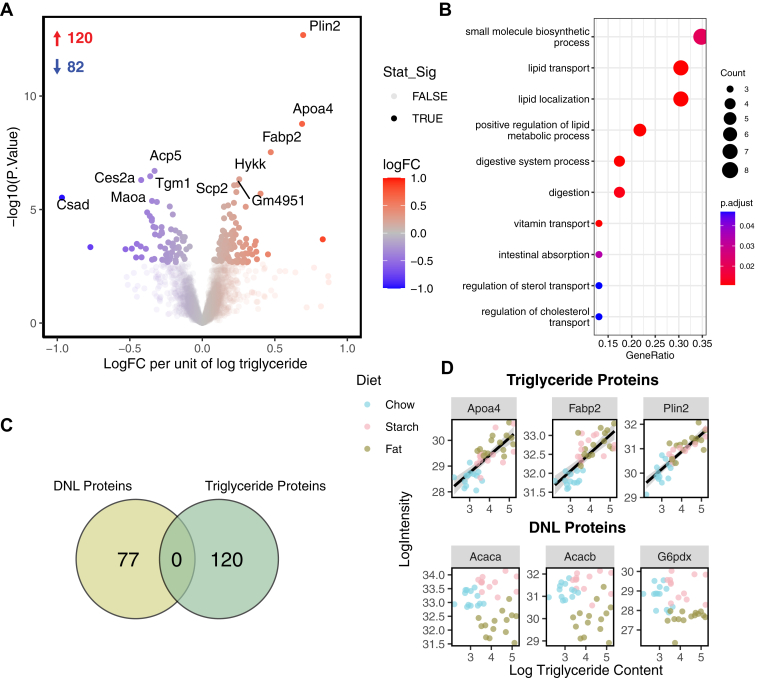
No overlap between proteins associated with DNL and TG content in the liver. A: Volcano plot of liver proteins that positively (red) or negatively (blue) associate with the liver TG content in mice. Transparent dots are proteins that did not reach the statistical significance threshold (FDR <0.05). B: Gene overrepresentation analysis (Gene Ontology-biological process [GO-BP]) of proteins that were significantly positively associated with liver TG content (FDR <0.01) over a background of all proteins quantified after filtering, color represents the adjusted *P* value of the term, dot size represents the counts within that term. C: Venn diagram showing no overlap between proteins that were significantly associated with DNL or TG content. D: Correlation between protein abundance and TG content for the three most significantly correlated proteins to TG content and DNL.

In a similar approach to above, we then correlated protein abundance with liver TG content in each mouse to assess if there was an overlap between proteins associated with liver DNL and TG storage. There were 120 proteins that had a significant positive correlation with liver TG content and 82 proteins with a significant negative correlation (FDR <0.05, Fig. 3A). The protein that displayed the tightest correlation with TG content was Plin2, a well-characterized hepatic lipid droplet protein (35). Pathways enriched from genes that were positively associated with TG content include lipid transport and localization (Fig. 3B). Interestingly, there was no overlap between proteins that positively correlated with DNL and those that positively correlated with TG content (Fig. 3C). Figure 3D depicts the top three TG-associated or DNL-associated proteins correlating protein abundance with TG content (Fig. 3D). There is a clear association between protein abundance and TG content in the TG-associated proteins that is not present in the top DNL-associated proteins.

### Large effect of nonpurified (standard laboratory chow) versus semipurified diet on the liver proteome

**Fig. 4:**
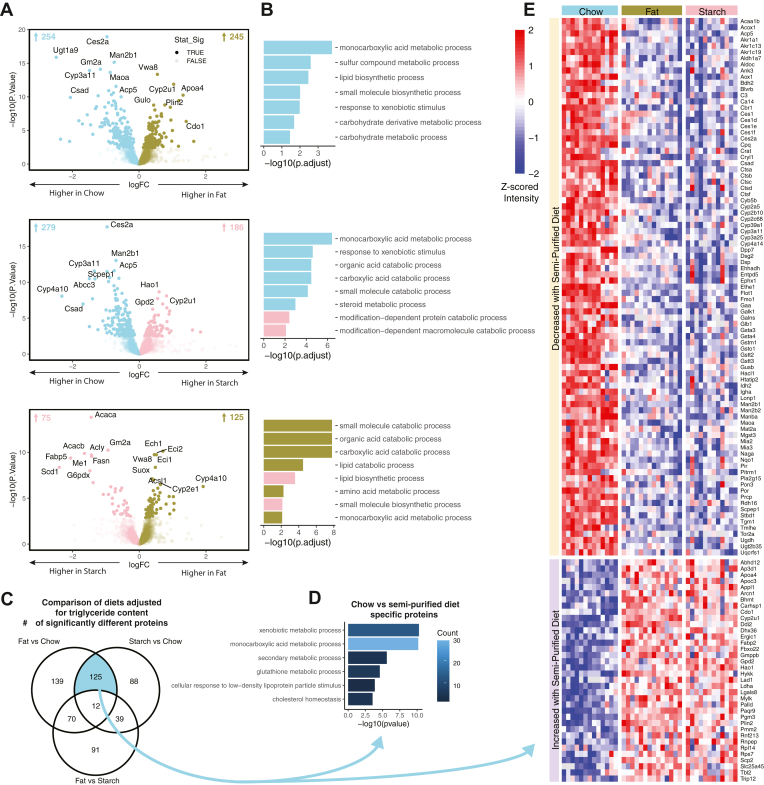
Large effect of chow versus semipurified diets on liver proteome. A: Volcano plots of significantly differentially abundant proteins between high-fat versus chow, high-starch versus chow and high-fat versus high-starch diets. Transparent dots are proteins that did not reach the statistical significance threshold (FDR <0.05). B: Gene set enrichment analysis (Gene Ontology-biological process [GO-BP]) of these contrasts colored for pathways that go up by this diet. C: Venn diagram showing overlaps between proteins that were significantly changed by the diet comparisons, corrected for liver TG content. The blue colored overlap represents proteins that are differentially abundant when comparing chow to both high-fat and high-starch diets but not differentially abundant when comparing high starch to high fat. D: Gene overrepresentation analysis (Gene Ontology-biological process [GO-BP]) of proteins specified in panel (C), over a background of all proteins quantified after filtering, color represents the count of differentially abundant proteins in this term. E: Heatmap of the proteins specified in panel (C), color indicates row scaled (z-scored) log2 intensity (semipurified/chow).

To complement our analyses of proteins that associate with continuous phenotypes, we next investigated the influence of the three different diets on the liver proteome. When comparing between chow and high-fat or high-starch diets, there were 498 and 463 proteins that were significantly differentially abundant, respectively (Fig. 4A). Pathway enrichment analysis identified “monocarboxylic acid metabolic process” and “response to xenobiotic stimulus” as pathways upregulated in the liver from chow-fed mice compared to either high-fat or high-starch diets (Fig. 4B). In comparison, there were far fewer proteins different between high-fat and high-starch-fed mice (200), with core DNL proteins being the highest upregulated proteins in high-starch mice (Acaca, Acacb, Me1, Acly, Scd1, and G6pdx) and beta-oxidation enzymes, the highest upregulated proteins in high-fat mice (Ech1, Eci1, and Eci2) (Fig. 3A). Correspondingly, pathway enrichment analysis identified pathways involved in lipid catabolism to be upregulated in high-fat mice and pathways involved in lipid biosynthesis to be upregulated in high-starch mice (Fig. 4B).

Due to the large effect of chow compared to the two semipurified diets (high fat and high starch), we next wanted to identify if there were proteins that were altered entirely by the comparison of semipurified versus nonpurified (chow) diet. To do this, we reanalyzed the dietary comparisons represented in Figure 4A; however, adjusted for liver TG content in the linear model. There were 124 proteins that were commonly differentially abundant between the high fat versus chow and high starch versus chow contrasts but were not differentially abundant when comparing the high-fat versus high-starch diets (Fig. 4C). Gene overrepresentation analysis of this group of proteins shows pathways enriched for xenobiotic metabolism and monocarboxylic acid metabolism as the most significant terms (Fig. 4D). Relative protein abundance between diets of the proteins in this group is depicted in Figure 4E, including multiple carboxylesterase (Ces1, Ces1d, Ces1e, Ces1f, and Ces2a), Cathepsin (Ctsa, Ctsb, Ctsc, Ctsd, and Ctsf), Cytochrome p450 (Cyp2b10, Cyp2c68, Cyp39a1, Cyp3a11, Cyp3a25, Cyp4a14, and Cyp2u1) and glutathione-*S*-transferase (Gsta3, Gsta4, Gstm1, Gsto1, Gstt2, Gstt3, and Mgst) enzymes, most of which are involved in phase I or II of the detoxification pathway or associated with autophagy.

### The effect of diet on the liver proteome is relatively stable between short- and long-term feeding

Finally, we wanted to investigate how similar, or different, diet-induced changes in the liver proteome were between short-term and long-term diet feeding. To do this, we utilized a separate cohort of mice that were fed the three test diets (chow, starch, and fat) for 30 weeks, where the high starch and fat groups were still fatter with higher liver TG levels compared to chow controls (supplemental Fig. S3) and compared the proteomic changes between the two cohorts. After filtering for missing values, there were 3,110 proteins that were quantified when combining the 4-week and 30-week diet-fed cohorts. When visualized by MDS plots, there were clear clusters by dietary group (most clear between chow and the two semipurified diets) and time on diet (supplemental Fig S1C).

**Fig. 5:**
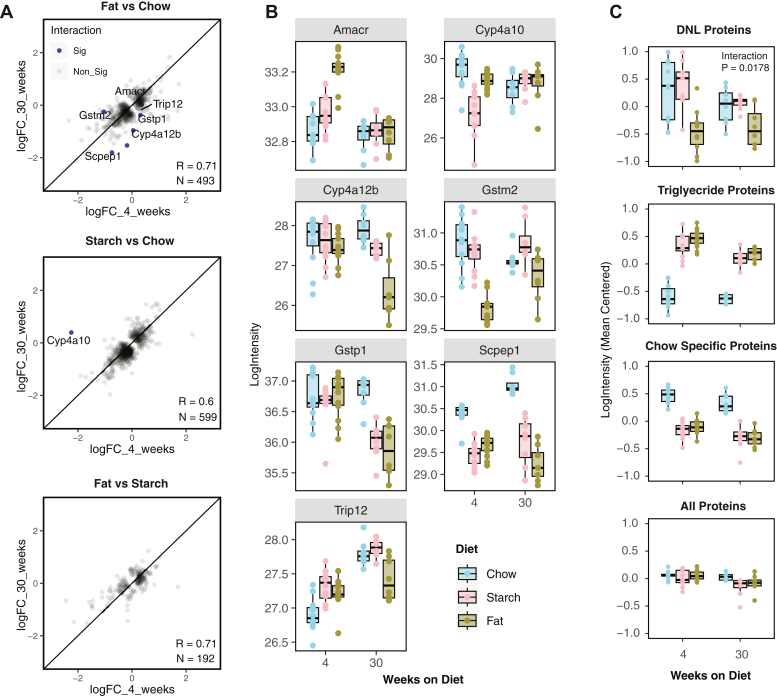
The effect of diet on the liver proteome is similar between short- and long-term diet feeding. A: Interaction between diet and weeks of feeding with logFC between diets after 4 weeks of feeding plotted on the *x*-axis and logFC between diets after 30 weeks of feeding plotted on the *y*-axis. Proteins considered statistically significant between diets by at least one of the two times are shown. Proteins that display a significant diet ∗ time of diet interaction are depicted in blue. B: Boxplots of the seven proteins that display a significant diet ∗ time of diet interaction. C: Proteins that were significantly positively associated with DNL or TG content or identified as chow versus semipurified diet specific (Fig. 4C) were mean centered and then averaged for each mouse and then analyzed by two-way ANOVA for a diet ∗ time of diet interaction.

Diet-induced changes in the liver proteome were remarkably consistent between 4 and 30 week-fed mice. The correlation coefficients for the logFC of proteins determined significant in at least one time point comparing 4 weeks and 30 weeks were between 0.6 and 0.71 (Fig. 5A). When testing for a diet–time interaction between the three diets, only seven proteins were significant and are displayed in Figure 5B, including two cytochrome P450 4A proteins (Cyp4a10 and Cyp4a12b) and two glutathione-*S*-transferase proteins (Gstm2, Gstp1), which may be involved in the metabolism of xenobiotics. When we looked at the groups of proteins found to be altered in the 4-week cohort in Fig. 2, Fig. 3, Fig. 4, we found that proteins in the DNL pathway, on average, displayed a significant diet–time interaction with 30-week-fed mice showing a smaller effect of diet on protein abundance (Fig. 5C). There was no significant diet–time interaction for TG-related proteins, chow specific proteins, or when all detected proteins were analyzed in this way.

## Discussion

In the current study, we utilized mice fed three different diets that differed in liver TG content and rate of DNL and performed proteomics to identify novel proteins that may be involved in the hepatic DNL pathway and to characterize the effect of short- or long-term feeding of these diets on the liver proteome. This approach differs from the commonly used knockdown or transgenic overexpressed mice that have been used to explore the regulation of DNL and has identified several proteins that may play a direct role in the regulation of hepatic DNL that are not canonical SREBP or ChREBP targets. Mice were housed at thermoneutrality to reduce the effect of cold stress on liver metabolism and to better model the human situation. This is because we have previously found mice housed at 22°C have a diet-specific increase in the abundance of DNL proteins in the liver compared to mice housed at thermoneutrality (36).

The protein that displayed the most significant positive correlation with the rate of hepatic DNL that is not known to be part of the DNL pathway was phosphodiesterase-related Protein (Pter). Pter has been recently identified as a taurine *N*-acetyltransferase/hydrolase, which catalyzes the N-acetylation of taurine with acetate and the reverse reaction (37). Correspondingly, Pter has been proposed to utilize excess N-acetyl taurine secreted from working muscle (38) and after alcohol consumption (39), to provide acetate as a substrate for hepatic DNL. Single nucleotide polymorphisms in Pter are associated with morbid obesity (40) and recently, Pter knockout mice were shown to have a small reduction in body weight gain when supplemented with taurine (37). Interestingly, flavin containing dimethylaniline monoxygenase 4 (Fmo4), which comes directly after Pter in correlation to rate of DNL, has also been linked with hepatic taurine metabolism potentially through the production of taurine from hypotaurine (41).

There were several SREBP2 targets involved in cholesterol synthesis (Lss, Fdps) that significantly correlate with hepatic DNL, which may indicate that cholesterol biosynthesis is upregulated as a biproduct of elevated DNL, potentially to support the increase in stored TG. This is in line with our previous liver lipidomic analysis, which showed mice fed a high-starch diet had increased cholesterol ester levels when compared to chow- and high-fat-fed mice (10). Caspase 3 (Casp3, also known as Cpp32), normally associated with apoptosis/cell death, was also positively correlated with DNL (Table 1). This protein has previously been reported to activate SREBP2 during apoptosis (42) but also in a cell death-independent manner (43). Our correlative data support the mechanistic studies from the literature, which Casp3 may be important in the transcriptional regulation of DNL proteins.

In this study, utilizing a mouse dietary model, proteins that correlated with liver TG content were distinct from proteins that associated with the rate of DNL, with classic lipid droplet proteins and lipoproteins (Plin2, Apoa4, and Fabp2) substantially upregulated. This is not an unexpected finding as the de novo synthesis of lipids and the storage/secretion of lipids are mechanistically separate pathways, and the latter includes lipid from dietary sources and from adipose tissue lipolysis. However, this does not mean that DNL is not a contributor to NAFLD. Our study showed mice fed a palatable high-starch diet displayed increased adiposity and liver fat content compared to chow mice (similar carbohydrate content of diet), which suggests that when coupled with calorie excess, DNL can be a major contributor to liver steatosis in mice. Our work (10, 44) as well as that of others (reviewed in Ref. (45)) suggests that increased hepatic DNL from a high carbohydrate diet during chronic positive energy balance may protect from some of the effects of ectopic lipid deposition on insulin sensitivity and glucose intolerance. This may be due to DNL producing palmitate in a well-regulated continuous process compared to the absorption of large amounts of dietary palmitate in a bolus, which may have adverse metabolic effects.

The diet-regulated liver proteome was remarkably similar between 4 and 30-week-fed mice with only a handful of proteins displaying a significant diet by time of feeding interaction. This suggests that most of the proteins quantified change rapidly in response to a new dietary composition rather than showing a progressive increase or decrease over time (such as diet-related changes in body weight/fat mass). Despite, 4-week and 30-week diet-fed mice showing overall similar liver protein abundance, there was a reduced abundance of proteins that associated with hepatic DNL in chow- and starch-fed mouse liver at 30 compared to 4 weeks suggesting that once enough fat is deposited, DNL may downregulate under conditions of high dietary carbohydrate.

When comparing between diets, we were interested to find that the biggest differences were between the nonpurified chow diet and either the semipurified high-starch or high-fat diet. This comparison remained when adjusting for liver TG content, suggesting that the sources and variety of dietary components have a similar or larger influence on the liver proteome than the macronutrient composition (percent of energy from fat, carbohydrate, or protein). Proteins that were altered by chow versus semipurified diets were involved in xenobiotic metabolism similar to a transcriptomic analysis of this comparison (46). It is likely that many of the proteins upregulated by a nonpurified diet in the liver are involved in the metabolism of less common dietary components, metabolites and contaminants that are either absent or not highly present in a semipurified diet. Polyphenols, including phytoestrogens, are derived from plants and are examples of non-nutrient compounds that may be present in brown chow and not in semipurified diets (47). Therefore, proteomic studies comparing a semipurified high-fat diet to a nonpurified chow control may identify proteins that are altered by the processing of the diet rather than the fat content or metabolic outcome of the diet as has been suggested (48, 49). In Figure 4 and the supplemental data, we provide a list of the liver proteins that were differentially abundant between chow and semipurified diets for use to screen potential hits when comparing between nonpurified and purified diets. Thus, our study reaffirms the growing consensus found in the literature (47, 48, 49) that the selection of an appropriate control diet is essential when trying to determine the molecular mechanisms altered by dietary intervention.

A limitation of the current study is that only one sex was used. Males have traditionally been the sex studied in the past in numerous research areas, including nutrition, obesity, diabetes, and aging (50). However, there is an increasing amount of evidence that the males and females have differences in numerous outcomes and are not more variable than males (51, 52). In the current study, one of the proteins that Fmo4, which is from a family of proteins that display large differences in liver abundance between sexes (53). Future experiments will elucidate the interactions between diet and sex on lipid metabolism in the liver. Another limitation of this study is that as there were small macronutrient differences between the chow and semipurified high-starch diet, we cannot definitively say that “chow- versus semipurified-specific proteins” isolated by the analysis in Figure 4 are due to the degree of dietary processing alone.

In conclusion, we identify liver proteins that positively correlate with hepatic DNL, including core enzymes in the DNL pathway. In addition, we found two novel proteins that have no previous characterized role in DNL, specifically Pter and Fmo4 that are involved in taurine metabolism, providing a potential link between these pathways. We find that the largest effect of diet on the liver proteome is when the nonpurified standard chow diet is compared with either of the semipurified diets and provide a list of proteins altered by the degree of dietary processing. Finally, we find that the diet-regulated liver proteome is remarkably similar between short- and long-term diet feeding indicating that the adaptation to new dietary components is rapid.

## Data availability

Mass spectrometry proteomics data have been deposited to the ProteomeXchange Consortium via the PRIDE partner repository with the dataset identifier PXD054323. The R code used for proteomics data analysis can be found at https://github.com/lewinsmall/Lipogenesis-Proteomics.

## Supplemental data

This article contains supplemental data.

## Conflict of interest

The authors declare that they have no conflicts of interest with the contents of this article.
