# Supplementary material for: Liver proteomics identifies a disconnect between proteins associated with de novo lipogenesis and triglyceride storage
Source: J Lipid Res. 2024 Oct 25;65(12):100687. doi: 10.1016/j.jlr.2024.100687 (PMC11626007; doi:10.1016/j.jlr.2024.100687)
Supplement: Supplemental Figs. S1–S3 [file mmc1.docx]

**Supplemental Figures**

**Liver proteomics identifies a disconnect between proteins associated with *de novo* lipogenesis and triglyceride storage**

Lewin Small^1,2*^, Tuong-Vi Nguyen^2^, Mark Larance^3^, Darren N. Saunders^3^, Andrew J. Hoy^3^, Carsten Schmitz-Peiffer^1,2^, Gregory J. Cooney^2,3^ and Amanda E. Brandon^1,2*^

^1^School of Life and Environmental Sciences, Charles Perkins Centre, Faculty of Science, The University of Sydney, NSW, Australia

^2^Garvan Institute, Sydney, NSW, Australia.

^3^School of Medical Sciences, Charles Perkins Centre, Faculty of Medicine and Health, University of Sydney, NSW, Australia.

**Figure S1: Multidimensional scaling (MDS) plots of proteomics data.** (A) MDS plot of 4 week fed mice before exclusion of muscle contaminated outlier samples circled in black, plotting dimension 1 again dimension 2 (B) MDS plot of 4-week fed mice after exclusion of outliers, plotting dimension 1 again dimension 2. (C) MDS plots of 4- and 30-week diet fed mice combined coloured by diet (top row) or weeks on diet (bottom row) plotting each combination of dimensions 1 to 3 (columns).

**Figure S2: Relationship between the proteins involved in DNL determined via mass spectrometry (MS) and western blotting (WB) or activity assay.** All proteins determined via MS were significantly positively correlated with either the protein levels determined via WB (A-D) or their activity (E-F). MS derived Abundance of Acaca and Acacb were compared to the same pan-ACC antibody WB quantitation. All data were expressed as log2 of the intensity (MS), abundance (WB) or activity. Data expressed as individual points. Correlations were analysed via linear regression with the significance and r^2^ value reported, n = 12-15. Raw WB images are contained in the supplemental data in (10).

**Figure S3: Physiological data from mice fed chow, a high starch or a high fat diet for 30 weeks**. (A) Body weight, (B) fat mass (at 16 weeks) and (C) liver triglyceride content (after cull at 30 weeks) was increased in high starch and high fat mice when compared to chow controls. Data presented as mean ± SEM. Analysed by individual one-way ANOVAs with Tukey’s multiple comparison tests to test between diets, n = 12-15. *p<0.05, ***p<0.001 ****p<0.0001 different to chow.
